# Defining reference genes in *Oryza sativa *using organ, development, biotic and abiotic transcriptome datasets

**DOI:** 10.1186/1471-2229-10-56

**Published:** 2010-03-31

**Authors:** Reena Narsai, Aneta Ivanova, Sophia Ng, James Whelan

**Affiliations:** 1 ARC Centre of Excellence in Plant Energy Biology, MCS Building M316 University of Western Australia, 35 Stirling Highway, Crawley 6009, Western Australia, Australia

## Abstract

**Background:**

Reference genes are widely used to normalise transcript abundance data determined by quantitative RT-PCR and microarrays. However, the approaches taken to define reference genes can be variable. Although *Oryza sativa *(rice) is a widely used model plant and important crop specie, there has been no comprehensive analysis carried out to define superior reference genes.

**Results:**

Analysis of 136 Affymetrix transcriptome datasets comprising of 373 genome microarrays from studies in rice that encompass tissue, developmental, abiotic, biotic and hormonal transcriptome datasets identified 151 genes whose expression was considered relatively stable under all conditions. A sub-set of 12 of these genes were validated by quantitative RT-PCR and were seen to be stable under a number of conditions. All except one gene that has been previously proposed as a stably expressed gene for rice, were observed to change significantly under some treatment.

**Conclusion:**

A new set of reference genes that are stable across tissue, development, stress and hormonal treatments have been identified in rice. This provides a superior set of reference genes for future studies in rice. It confirms the approach of mining large scale datasets as a robust method to define reference genes, but cautions against using gene orthology or counterparts of reference genes in other plant species as a means of defining reference genes.

## Background

The analysis of gene expression, or more correctly transcript abundance, is widely carried out in a variety of laboratories in various disciplines. Northern blotting, quantitative RT-PCR (QRT-PCR) and microarray approaches are commonly used to assess transcript abundance. All these approaches need a standard or reference for comparison, so that the changes observed can be attributed to a biological process rather than an artefact of the particular technique used [1,2]. The use of northern blotting often involves the use of equal RNA (total or mRNA) loading as a reference point. Although this can lead to errors, the variability of many steps in northern blotting means that northern blots are generally only used to assess large changes in transcript abundance. In contrast, microarray analysis assesses the transcript abundance of tens of thousands of genes, thus it has required the application of statistical methods to normalise the distribution of signals and also requires correction for large samples sets, so called false discovery rate correction [3,4]. For QRT-PCR analysis, house-keeping or reference genes can be used as a standard and by definition; the transcript abundance of this gene should not change under the experimental conditions being studied.

The definition of reference genes is important as the use of common sets of reference genes by scientists allows direct comparisons between studies. The benefits of comparing transcripts abundance datasets between a variety of studies is best exemplified with microarray studies, where the predominant use of a single robust platform for studies in *Arabidopsis thaliana *has led to the development of a number of databases where *in silico *or digital northern analyses can be carried out. Thus, databases such as Genevestigator [5] and the Botany Array Resource (BAR) [6] are just two examples that provide a valuable resource for researchers to obtain information of transcript abundance patterns for genes of interest.

QRT-PCR is often used to validate transcriptome data obtained from array studies or is used in more directed studies where the transcript abundance of a limited number of genes is analysed. Increasingly large scale studies encompassing several hundred to thousands of genes are also analysed by QRT-PCR and represent an important resource to the scientific community, e.g. expression profiling of transcription factors [7-9]. Thus, accurate reference genes are required to interpret such data. In an Arabidopsis study that defined stably expressed genes under a wide variety of conditions and organs, a "superior set" of reference genes were identified that are widely used in QRT-PCR studies in Arabidopsis [10]. An alternative approach to define reference genes is the use of various statistical tests that essentially rank the variability of transcripts abundances for sets of genes that are analysed [1]. Bestkeeper [11], Norm-Finder [12] and geNORM [13] are examples of such widely used programs, albeit their use is limited to some extent in studies with plants [2].

A variety of studies in different plant species have defined reference genes [2]. Many studies selected a number of potential reference genes based on what is used in other plant species, and tested changes in transcript abundance, using statistical algorithms outlined above to test for variations in different organs or environmental conditions, to determine their suitability as reference genes [14-17]. All these studies have defined reference genes, but the limited number of conditions tested and the lack of genome wide searches for superior reference genes means that these sets may not represent the best reference genes under a wide variety of conditions. The ability of software programs to define variations in gene expression is limited by the input data. However, it is desirable to define reference genes that are stable in transcript abundance under as many conditions as possible and analysing as many genes in the genome as possible.

*Oryza sativa *(rice) represents an important model plant [18] and as a crop, provides 21% of the calorie needs of the world's population (and up to ~75% for the population of south east Asia [19]. As such, it is the focus of intense research by a wide variety of researchers. One of the fundamental problems facing researchers carrying out gene expression studies is the use of control or reference genes that should not change, preferably under all experimental conditions. Reference genes in rice have been proposed by testing commonly used reference genes in plants and orthologues of reference genes that have been defined as in Arabidopsis [7,20]. It is unclear under how many different parameters these genes are appropriate reference genes and also if superior reference genes could be defined using a genome wide approach as previously carried out in Arabidopsis [10].

In order to define suitable reference genes in rice in an objective manner, a similar procedure to that used to define reference genes in Arabidopsis was undertaken [10]. We collated 373 Affymetrix genome arrays from rice that encompassed tissue, abiotic, biotic and hormonal parameters to define a set of 151 probesets that were stably expressed under all conditions. Of these, 12 genes were chosen as reference genes and validated using QRT-PCR, for different tissues and under stress. In this way, a superior set of reference genes for rice was identified that are suitable for organ, development and stress based experiments.

## Results and Discussion

### Selection of transcriptome datasets

To meet the criteria for a suitable reference gene, the transcript must be detected in all organs, developmental conditions and under a variety of stress conditions. In order to identify genes that fulfilled these criteria, all transcriptome data available for rice on the Affymetrix platform (August 2009) was utilised. Apart from being widely used, it contains a variety of datasets that can be analysed together on a common platform. Thus, data from 373 microarrays were analysed together from experiments encompassing tissue development sets (embryo, endosperm, dry seed, germinating seed, coleoptiles, leaf, apical meristem, root, stigma, ovary, and inflorescence), abiotic stress (cold, heat, drought, salt, nutrient and physical), biotic stress (fungal, parasite, viral and bacterial) and hormone treatments are represented (Table 1). Additionally, as the experiments presented in these datasets have been performed in a variety of laboratories using different varieties of rice, it is likely that genes defined as not changing in expression are more likely to be robust.

**Table 1:** Overview of experiments involving 373 Affymetrix rice genome microarrays used for the global analysis in this study.

Sample description	Ref	GEO/other accession	Reps	Arrays	Tissue
*DEVELOPMENT/TISSUE*					

Dry seed and aerobic germination (up to 24 h) cv. Amaroo	[25]	E-MEXP-1766	3	15	Dry and germinating seed
Dry seed and anaerobic germination (up to 24 h) and switch conditions cv. Amaroo	[21]	E-MEXP-2267	3	36	Imbibed seed
Aerobic and anaerobic grown coleoptiles cv. Nipponbare	[27]	GSE6908	2	4	Coleoptile
Embryo, endosperm, leaf and root from 7-d seedling, 10-d seedling cv. Zhonghua	[28]	GSE11966	2	10	Embryo, endosperm, leaf and root from 7-d seedling, 10-d seedling
Stigma, Ovary+7 single arrays cv. Nipponbare	[29]	GSE7951	1-3	13	Stigma, ovary+7 single arrays
Mature leaf, young leaf, semi apical meristem, inflorescence, seed cv. IR64	[30]	GSE6893	3	45	Mature leaf, young leaf, semi apical meristem, inflorescence, seed

*ABIOTIC STRESS*					

Drought, salt, cold stress cv. IR64	[30]	GSE6901	3	12	Seedling
Heat stress cv. Zhonghua	[31]	GSE14275	3	6	Seedling
Salt stress on 2 cultivars; indica, FL478 (salt tolerant), indica, IR29 (salt sensitive)	[32]	GSE3053	3	11	Crown and growing point
Salt stress on 4 cultivars; japonica, m103 (salt sensitive), indica, IR29 (salt sensitive), japonica, Agami (salt tolerant), indica, IR63731 (salt tolerant)	[33]	GSE4438	3	24	Panicle initiation stage
Salt stress on root using 4 cultivars; FL478 (salt tolerant), IR29 (salt sensitive), IR63731 (salt tolerant), Pokkali (salt tolerant)	Not found	GSE14403	3	23	Root
Fe and P treatments cv. Nipponbare	[34]	GSE17245	2	16	Root
Arsenate treatment cv. Azucena	[35]	GSE4471	3	12	Seedling
Physical stress at roots tips cv. Bala	[35]	GSE10857	3	12	Root tip

*BIOTIC STRESS*					

*S.Hermonthica *plant parasite infection cv. Nipponbare (resistant), IAC165 (susceptible)	[36]	GSE10373	2	24	Root
*M.grisea *blast fungus infection cv. Nipponbare	[37]	GSE7256	2	8	Leaf
Rice stripe virus infection cv. WuYun3, KT95-418	Not found	GSE11025	3	12	Seedling
Infection with bacteria *X.Oryzae *pv. oryzicola and oryzae cv. Nipponbare	Not found	GSE16793	4	60	Whole-plant tissue

*HORMONE TREATMENT*					

Cytokinin treatment on root and leaf cv. Nipponbare	[38]	GSE6719	3	24	Root, 2-week old seedlings
Indole-3-actetic acid and benzyl aminopurine treatment cv. IR64	[39]	GSE5167	2	6	Seedling

The microarray experiments are classified as development/tissue, abiotic stress, biotic stress or hormone treatment respectively, depending on the purpose of the experiment. For each microarray dataset; the sample/experimental description, the respective cultivar (cv.), the corresponding publication (Ref - where available), public Gene Expression Omnibus (GSE) identifier or MIAME Genexpress identifier (E-MEXP), the number of biological replications carried out (Reps), the number of microarrays carried out in that experimental dataset and the tissues analysed are shown.

### Global analysis of transcriptome datasets

**Figure 1:**
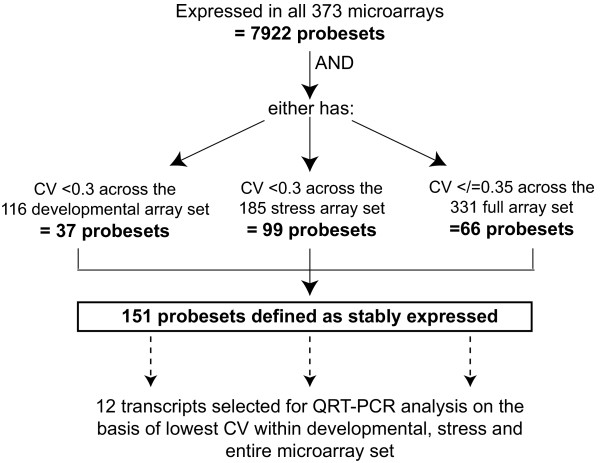
**Schematic of selection criteria for stably expressed genes and reference genes selected for QRT-PCR validation**. CV = coefficient of variance.

In order to analyse these multiple global rice transcriptome data in a comparable way, all arrays were normalised in the same way (materials and methods) and present/absent calls were determined MAS5.0 normalisation. The genome was defined as the 57,302 probesets targeted to *Oryza sativa*, thus the 81 probesets designed for the bacterial/phage controls were not included. The normalised data from all 373 microarrays (Table 1), representing 136 biological samples were collated together and a probeset was considered to be expressed in a particular tissue/sample if all replicates for every sample showed statistically significant present calls (p < 0.05). This cut-off method has previously been used as a way of present/absent determination [10,21]. Using this principle, the expression for each probeset across all microarrays could be determined. Nearly eight thousand (7,922) probesets were detected in all 373 microarray samples, thereby fulfilling the first criterion for defining reference genes (Figure 1).

### Selection of reference genes

The GC-RMA normalised data for all microarrays with publically available CEL files (331 microarrays; Table 1) was used to calculate the mean, standard deviation (SD) and coefficient of variance (CV; CV = SD/mean) for all 7,922 probesets, where a low CV is indicative of lower variation. This was followed by selection process undertaken to determine which of these genes were suitable as reference genes (Figure 1). Only 151 of the 7,922 probesets were defined as stably expressed across the developmental, stress and/or entire dataset (Figure 1).

**Figure 2:**
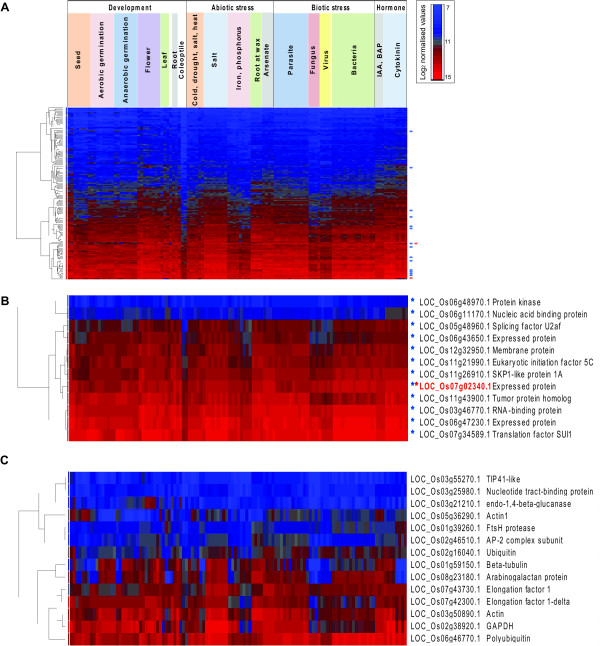
**Analysis of stably expressed genes**. **A) **Average linkage hierarchical clustering of the group of 151 probesets, based on CV criteria described in Figure 1. The genes are on the y-axis and the samples on the x-axis. The details of the treatments are outlined in Table 1. The scale is log2 normalised values where blue is low levels of transcript abundance and red is high levels of transcript abundance. Genes indicated by blue asterisk denotes novel reference genes indentified in this study, while red asterisk indicates genes previously defined as stably expressed in other studies [8,22]. **B) **The probesets indicated by blue asterisk (*) in A, were independently hierarchically clustered and analysed by QRT-PCR. **C) **Average linkage hierarchical clustering of the previously suggested/commonly used reference genes. The variation in transcript abundance across the various parameters is evident by the variation in colour intensity from left to right.

In order to visualise the expression of these 151 probesets, the log_2 _normalised values were hierarchically clustered and as expected, stable expression profiles were observed across the tissue development, stress and hormone microarray experiments (Figure 2A). Only 2 of these genes, LOC_Os07g02340.1 and LOC_Os03g05290.1, have been previously identified as stably expressed, with the former gene identified in a previous rice study [22], and the latter based on orthology with an Arabidopsis reference gene [7] (Figure 2B, red asterisk). A selection of 12 genes that showed stable expression across the microarrays (Figure 2B) were analysed further by QRT-PCR (Genes 1-12; Table 2). These 12 genes were selected on the basis of their CV and included; 2 transcripts with the lowest CV calculated across the stress microarray set (Genes 1-2), 2 transcripts with the lowest CV across the developmental set (Genes 3-4), 3 transcripts with the lowest CV across the entire microarray set (Genes 5-7) and the remaining 4 genes were randomly selected from the 66 probesets with low CV values (</=0.35; Genes 8-12) from the entire microarray set (Figure 1 and 2B; Table 2).

**Table 2:** The list of reference genes for rice, defined in this and previous studies.

Gene	Probe Set Identifier	TIGR Identifier	Description	Mean	SD	CV	MV	Source
1	Os.10676.1.S1_a_at	LOC_Os06g11170.1	Nucleic acid binding protein	991.9	210.2	**0.21**	**0.25**	This study
2	Os.8912.1.S1_at	LOC_Os06g48970.1	Protein kinase	453.3	96.8	**0.21**	**0.50**	This study
3	Os.6.1.S1_a_at	LOC_Os11g43900.1	Tumor protein homolog	13137.5	3692.7	**0.28**	**0.66**	This study
	Os.6.1.S1_x_at		Tumor protein homolog	13870.8	3368.4	**0.24**		This study
-	Os.12625.2.S1_x_at	No TIGR identifier	NA	18285.5	4473.7	**0.24**	**-**	This study
4	Os.12237.2.S1_a_at	LOC_Os06g47230.1	Expressed protein	18251.2	4481.0	**0.25**	**0.30**	This study
	Os.12237.1.S1_a_at		Expressed protein	22019.9	5294.2	**0.24**		This study
5	Os.46231.2.S1_x_at	LOC_Os03g46770.1	RNA-binding protein	17176.5	4280.7	**0.25**	**0.68**	This study
	Os.46231.1.S1_a_at		RNA-binding protein	22461.1	5636.0	**0.25**		This study
6	Os.6860.1.S1_at	LOC_Os11g21990.1	Eukaryotic initiation factor 5C	6969.6	1967.0	**0.28**	**0.54**	This study
7	Os.7945.1.S1_at	LOC_Os07g34589.1	Translation factor SUI1	24678.2	7030.8	**0.28**	**0.61**	This study
**8**	Os.12409.1.S1_at	LOC_Os07g02340.1	Expressed protein	11392.3	3488.8	**0.31**	**0.44**	This study
9	Os.37924.1.S1_x_at	LOC_Os11g26910.1	SKP1-like protein 1A	8488.5	2713.8	**0.32**	**0.85**	This study
10	Os.12382.1.S1_at	LOC_Os12g32950.1	Membrane protein	6550.4	2258.4	**0.34**	**0.59**	This study
11	Os.8092.1.S1_at	LOC_Os05g48960.1	Splicing factor U2af	4051.7	1403.7	**0.35**	**0.49**	This study
12	Os.12151.1.S1_at	LOC_Os06g43650.1	Expressed protein	4504.6	1581.7	**0.35**	**0.39**	This study
13	AFFX-Os-actin-3_s_at	LOC_Os03g50890.1	Actin	9556.3	5719.5	**0.60**	**0.97**	[7]; commonly used reference gene
14	Os.11355.1.S1_at	LOC_Os05g36290.1	Actin1	1842.8	1471.3	**0.80**	**0.79**	[7]; commonly used reference gene
15	Os.9504.1.S1_at	LOC_Os07g38730.1	Alpha-tubulin	5400.3	3466.6	**0.64**	**0.76**	[7]; commonly used reference gene
16	Os.10139.1.S1_s_at	LOC_Os06g46770.1	Polyubiquitin	15085.3	6524.3	**0.43**	**0.47**	[7]; commonly used reference gene
17	Os.7899.1.S1_at	LOC_Os02g16040.1	Ubiquitin	2598.8	1135.4	**0.44**	**0.63**	[20]; commonly used reference gene
18	Os.22781.1.S1_at	LOC_Os02g38920.1	GAPDH	11640.8	8346.8	**0.72**	**1.09**	[20]; commonly used reference gene
19	Os.10158.1.S1_at	LOC_Os07g43730.1	EF1	5619.9	2549.3	**0.45**	**0.52**	[20]; commonly used reference gene
20	Os.10385.1.S1_at	LOC_Os03g55270.1	TIP41-like	482.7	274.5	**0.57**	**0.42**	[7]
21	Os.5500.1.S1_s_at	LOC_Os08g23180.1	Arabinogalactan protein	4957.5	3114.1	**0.63**	**0.90**	[22]
22	Os.12835.2.S1_a_at	LOC_Os07g42300.1	EF1d	6073.3	3003.7	**0.49**	**0.82**	[22]
23	Os.19618.1.S1_at	LOC_Os01g39260.1	FtsH protease	1487.4	725.5	**0.49**	**0.57**	[22]
24	Os.7952.1.S1_at	LOC_Os03g25980.1	Nucleotide tract-binding protein	607.8	241.8	**0.40**	**0.56**	(Orthologue) [10]
25	Os.22806.1.S1_s_at	LOC_Os02g46510.1	AP-2 complex subunit	1550.2	744.5	**0.48**	**0.64**	(Orthologue) [10]
26	Os.13910.1.S1_at	LOC_Os03g21210.1	endo-1,4-beta-glucanase	900.7	1063.3	**1.18**	**0.72**	(Orthologue) [10]

The gene number, Affymetrix probeset identifiers, TIGR identifiers, gene descriptions (TIGR), mean expression and standard deviation (SD) based on GC-RMA normalised data. The coefficient of variance (CV) is also indicated for each probeset/gene. The M values calculated based the QRT-PCR data; using geNORM software is also shown. Source indicates the studies from which these genes were selected.

Closer analysis of these 12 genes reveals that the genes encoding, a 3-phosphoinositide-dependent protein kinase-1 (LOC_Os06g48970.1) and a nucleic acid binding protein (LOC_Os06g11170.1) showed stable, moderate expression levels across the stress microarray set (Genes 1-2 in Table 2; Figure 2B). While the genes encoding a tumor protein homolog (LOC_Os11g43900.1) and translation initiation factor SUI1 (LOC_Os07g34589.1) showed stable expression across the developmental and entire microarray sets respectively (Genes 4, 7 in Table 2; Figure 2B). As would be expected, it can be seen that many of these stably expressed genes are involved in core cellular functions such as mRNA splicing and translation initiation (Genes 1-12 denoted by blue asterisks in Figure 2A; 2B; Table 2).

**Figure 3:**
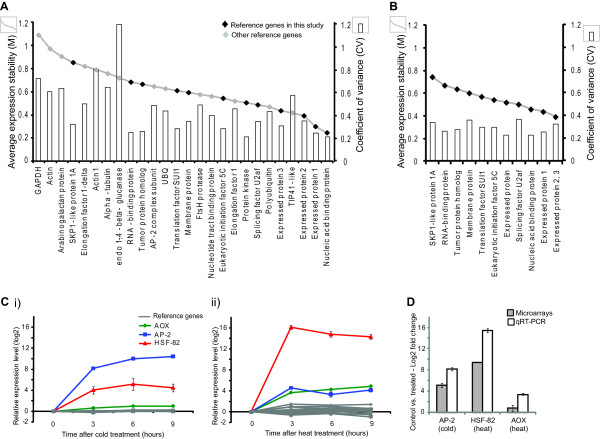
**QRT-PCR validation of proposed reference genes and comparison to previously suggested/commonly used reference genes**. **A) **geNORM output using QRT-PCR data showing average expression stability values of all commonly used and novel reference genes, lower M value indicates greater stability. The coefficient of variance for each gene across all the microarrays is also shown, lower CV indicates greater stability. Genes with low M value and low CV are the most stable. Genes not expressed in all microarrays are indicated with an asterisk (*). **B) **Transcript abundance of the 12 reference genes identified in this study (indicated in grey) and AP-2, HSF-82 and AOX in shoots from the (i) cold and (ii) heat treated (as indicated) seedlings over time. **C) **Comparison of the change in AP-2, HSF-82 and AOX transcript abundance (log_2 _fold change) in the leaves from the 3 h cold and heat treated (as indicated) seedlings compared to the control seedlings using microarrays and QRT-PCR.

In order to compare the reference genes defined in this study with the expression of some genes defined as stably expressed in these previous studies [7,10,22], 14 genes commonly used reference genes were visualised in the same way across the microarrays (Figure 2C) and the mean, SD and CV for each was also calculated (Genes 13-26; Table 2). It can be seen that there is a large amount of variation in transcript abundance for many of the previously proposed stably expressed genes as well as the typical reference genes, such as those encoding Actin and ubiquitin (Figure 2C; high CVs in Table 2). It is particularly evidenced that beta-tubulin transcript expression is variable under bacterial and parasite infection respectively (Figure 2C). Although the heatmap visualisation of the expression for the nucleotide tract-binding protein (LOC_Os03g25980.1) and TIP41-like protein (LOC_Os03g55270.1) appears unchanging (Figure 3 - top 2 genes), it can be seen that the CVs for both of those genes is over 0.4 indicating a higher level of variation in expression (Table 2).

### Validation of reference genes in quantitative RT-PCR in tissue and stress samples

In order to confirm stable expression of the reference genes identified in this study primers were designed to 26 genes,12 stably expressed genes identified in this study and 14 previously suggested reference genes (Table 1, Additional file 1, Table S1). The stability of transcript abundance of these genes was analysed by QRT-PCR across 15 different samples from a variety of developmental (dry seed, imbibed seed, leaf and roots from young and old plants) and stress treated tissues (shoots from cold treated and heat treated young seedlings over time; Materials and methods). High quality total RNA was isolated from these samples and reverse transcribed to generate cDNA. The same cDNA pool from each of the samples was used to measure the transcript abundance by QRT-PCR, with melt curve analysis for each gene confirming primer specificity.

The geNORM v3.5 software was used to analyse the expression stability for the reference genes analysed by QRT-PCR from the 12 tissue samples (Additional file 1, Table S1) [13]. This software allows calculation of a gene stability measure (M) value for all the genes analysed, where genes with the lowest M value shown the most stable expression (Figure 3A). Authors of the geNORM software suggest using the 3 most stable genes (3 lowest M values) as the most appropriate reference genes [13]. It can be seen that even when commonly or previously suggested reference genes and the novel reference genes from this study are analysed together, all 3 of the most stable genes are the novel reference genes identified in this study (Figure 3A). It is important to note that this M value is only calculated based on data from the limited number of samples that were analysed by QRT-PCR, thus not representing the wide variety of tissues/treatments analysed by microarrays. Therefore, in order to visualise the variation in expression across in the microarrays in parallel, the CV values for each gene was also plotted with the M values, where a lower CV value indicates greater stability. In this way, the most stable genes were identified as those with both low M and CV values. In this combined analysis, the 12 genes chosen all outperformed previously used reference genes, particularly in terms of having a lower CV (Figure 3A), the genes indicated with a black diamond all had lower CV values as indicated by the bar graph, with a gene encoding a nucleic acid binding protein (LOC_Os06g11170.1) apparently the most stable (Figure 3A).

To further test the stability of the reference genes defined in this study, the expression of the 12 novel reference genes defined in this study were analysed independently by geNORM for the samples analysed by QRT-PCR (Figure 3B and 3C). Overall, it can be seen that the most stable genes had low M values as well and low CV values, indicating stable expression (Figure 3B). Furthermore, the geNORM pair-wise analysis to determine the number of control genes recommended for use in normalisation [13], revealed that 2 or even one gene is stable enough for accurate normalisation, however 2 genes is recommended for more robust normalisation (V < 0.15; Additional file 2, Figure S1) [13]. Using QRT-PCR analysis, we also compared the expression of these 12 reference genes to 3 heat or cold responsive genes including, an Apetala type transcription factor (*AP2*), a heat shock responsive factor (*HSF-82*) and alternative oxidase (*AOX*) over time under i) cold or ii) heat conditions respectively (Figure 3C). It can be observed that under cold treatment, all 12 reference genes show very stable expression over time (Figure 3Ci). Similarly, despite slight variation of some genes under heat conditions, it is evidenced that overall, these genes are also stably expressed over time following heat treatment (Figure 3Cii). In addition, the observed induction of *AP2 *and *HSF-82 *under cold and heat treatment, confirmed the success of the respective treatments (Figure 3C). Furthermore, comparison of this induction (at 3 h) to the induction observed from the analogous microarray data, showed that normalisation of the QRT-PCR data using the reference genes defined in this study resulted in comparable increases to those seen using the microarray data (Figure 3C).

### Comparison to previous studies and other expression platforms

A large-scale study of reference genes in Arabidopsis revealed superior reference genes using Affymetrix microarray data [10]. Using the Inparanoid orthologue output [23] for Arabidopsis and rice, it was seen that only 15 rice orthologues of the 30 novel Arabidopsis reference genes were also expressed across all the microarrays in this study and 3 of these were randomly selected for further analysis by QRT-PCR (Genes 24-26; Table 2). Notably, only 1 gene (LOC_Os03g05290.1) encoding an aquaporin TIP protein, was seen to be stably expressed i.e. one of the 151 stably expressed in this study (red asterisk only; Figure 2A). It may be noted that the overall CV values are higher in this study compared to the CV values calculated in the Arabidopsis study [10]. The main reason for this is likely to be due to significant differences in the variability of the input data from both studies. That is, the Arabidopsis reference gene study used microarray data generated from only 7 studies using a large number of microarrays each e.g. 237 microarrays in the single developmental study [10], whilst this study involved analysis of microarrays from 20 studies carried out in different laboratories, using between 4 and 60 microarrays in each.

Previous studies in rice have examined reference genes using QRT-PCR analysis, however these only involved analysis of a small number of commonly used reference genes such as Actin, Actin1, alpha and beta tubulin, polyubiquitin, ubiquitin, GAPDH and elongation factor 1 in up to 25 samples, under a limited range of conditions [7,20]. Analysis of these genes in the context of this study (Genes 13-20; Table 2) revealed that some of these were not detected as expressed in one or more tissue/stress microarray experiments, notably, this included *Actin1 *(LOC_Os05g36290.1; Gene 14 in Table 2) which was not expressed in all 3 biological replicates of the semi apical meristem (GSE6901) (Figure 2C). Similarly, a recent study in rice defined a set of 248 stably expressed genes across 40 developmental tissues that were analysed using Yale/BGI oligonucleotide microarrays [22]. Only 61 of these genes were found to be expressed across all the microarrays analysed in this study, nevertheless 3 of these were randomly selected for further analysis by QRT-PCR (Genes 21-23; Table 2). Notably, one of the 61 genes (LOC_Os07g02340.1) encoding an "expressed protein" was also found to fulfil all the criteria outlined in Figure 1, and showed stable expression across all the samples analysed in the present study (Gene 8 in Table 2; denoted by red and blue asterisk in Figure 2A and 2B).

**Figure 4:**
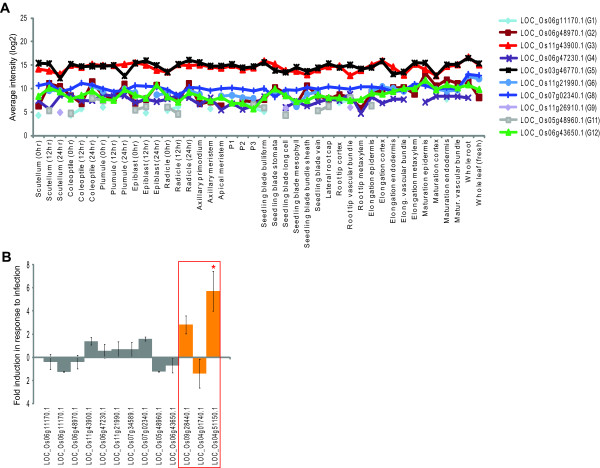
**Expression of the proposed reference genes using other platforms**. **A) **Transcript abundance levels for the 12 proposed references genes based on data using the Rice Yale/BGI oligonucleotide microarray. The average intensity (using >2 replicates) were log2 transformed and visualised across the tissues analysed in a previous study [22]. **B) **Change in transcript abundance for the 12 proposed reference genes (grey) and AP-2, HSF and AOX (red box) following infection with hemibiotrophic fungus *Magnaporthe oryzae *[24]. Absolute fold-change values are shown (+/- standard error). Significant changes (t-test, p < 0.01) are indicated by a red asterisk.

In order to test the robustness of expression stability for the 12 reference genes identified in this study, two different approaches were undertaken. Firstly the expression patterns of these 12 genes were examined on other expression platforms, specifically the BGI/Yale oligonucleotide and Agilent microarray platforms. Overall a stable expression pattern was observed for all genes examined, with the most stable expression particularly evidenced for LOC_Os11g43900.1, LOC_Os03g46770.1 and LOC_Os07g02340.1 using the Yale oligonucleotide microarrays (Figure 4A). Notably, the latter gene was also grouped within the 248 stably expressed genes defined previously identified [22], thus complementing the identification of this gene in the presented study. Similarly, the 12 reference genes identified in this study were also examined for changes in expression following infection with hemibiotrophic fungus *Magnaporthe oryzae *[24]. In this study, Agilent Arrays (G4138A) were used for global transcriptomic analysis following infection [24]. The expression of all 12 genes were not found to significantly differ (Students t-test, p < 0.01) following infection (Figure 4B). However, given that this experiment involved stress treatment; AP-2, HSF-82 and AOX expression were also examined following infection and it was observed that AOX was significantly up-regulated (p < 0.01) following infection (Figure 4B). AOX is a known stress responsive gene [25]. Thus the reference genes defined are stable even under biotic stress stimulation, in addition to the abiotic treatments carried out as described above.

## Conclusion

The use of the large datasets of rice microarray data has provided identification of sets of genes that are stably expressed under a wide variety of parameters. Although microarray platforms were not designed to be quantitative, direct comparison of over 1000 QRT-PCR assays with microarray data has revealed a high degree of correlation [26]. This is consistent with the use of microarray data to define superior reference genes as outlined here, and previously in Arabidopsis [10]. Based on these principles, we suggest the use of one or more of the novel reference genes presented in this study for the normalisation of rice microarray or QRT-PCR data. However although the reference genes identified in this study are stable under a wide variety of parameters, such as developmental, tissue and various stresses, it is essential that each study validate the stability of the selected reference gene(s) to achieve the systematic validation of reference genes that is required to compare different studies [2].

## Methods

### Analyses of all publically available rice microarrays

To compile the entire publically available Affymetrix rice microarray (as at 1^st ^August 2009), all experiments containing CEL files were downloaded from the Gene Expression Omnibus within the National Centre for Biotechnology Information database or from the MIAME ArrayExpress database http://www.ebi.ac.uk/arrayexpress/. The GSE or EXP numbers for the respective rice studies are shown in Table 1. There was a total of 373 microarrays for which there was either MAS5.0 data available, thus all of these were used for present/absent determination in defining the list of 7,922 probesets expressed in all microarrays. However of these 373 microarrays, 7 had no biological replicates and 35 did not have available CEL files, thus the remaining 331 microarrays were used to carry out further normalisation (GC-RMA) and calculation of mean, standard deviation and coefficient of variance (CV). This allowed analysis of 117 tissues/conditions, with a minimum of 2 biological replicates. The 117 included 41 organ/developmental tissues, 65 samples within abiotic and biotic stress experiments and 11 samples within hormone treatment experiments.

All raw intensity CEL files were imported into Avadis 4.3 (Strand Genomics) and the standard MAS5.0 normalisation was first carried out in order to determine present/absent/marginal calls for each probeset. For all 331 microarrays with available CEL files (and carried out for biological replicates), GC-RMA normalisation was carried out. The mean expression, SD and CV (=SD/mean) was then calculated for each of the 7,922 probesets across the developmental set, stress set and entire dataset (which included the hormone experiments). On the basis of CV cut-offs, the list of 151 probesets was generated (Figure 1). The averaged log GC-RMA normalised values for these 151 probesets, across the developmental tissues, stress and hormone treatment experiments were hierarchically clustered using average linkage on Euclidean distance. The clustering analysis and heatmap generation was carried out using Partek Genomics Suite, version 6.3 (Partek). For the Agilent microarray comparison, data was retrieved under the accession GSE8518 from the Gene Expression Omnibus within the National Centre for Biotechnology Information database.

### Analysis of orthologues

The InParanoid: Eukaryotic Orthologue Groups database (version 7.0) was used to analyse all orthologues between rice and Arabidopsis [23]. The orthologous group file was downloaded for the whole-genome comparison of rice versus Arabidopsis. This produced information for orthologues identified by TIGR identifiers for rice and AGIs for Arabidopsis.

### Stress treatments, tissue collection and RNA isolation

In order to analyse the expression of all the genes in Table 2, a selection of tissues were collected across different developmental stages/tissues and under different stress conditions in wild type rice, cv. Amaroo. In order to analyse different developmental tissues; embryos were extracted from dry seed, seeds imbibed for 24 h with oxygen gas (24 h A), seeds imbibed for 24 h in the absence of oxygen gas i.e. in the presence of nitrogen gas (24 h N), seeds imbibed for 24 h under nitrogen gas and switched to oxygen gas for 3 h (27 NA), leaf and root tissues from 2-week old seedlings and 3 month old plants. Furthermore, to examine the effects of abiotic stress, 2-week old seedlings were transferred to 4°C and 42°C for cold and heat treatment respectively over a 9 h time course, whilst the controls remained at a constant temperature of 30°C. All 15 tissue samples were analysed using three biological replicates, the RNA was isolated using the Qiagen RNeasy Plant RNA isolation kit and DNase treated using both the Qiagen on-column DNase digestion as well as the Ambion Turbo DNase treatment exactly as carried out in Howell et al., 2009 [27].

### QRT-PCR analysis

Details of the primer sequences and amplicon lengths for each of the genes are shown in Additional file 1, Table S1. The transcript abundance for each gene was analysed using the SYBR green I master (Roche, Sydney) with the Roche LC480. Each sample was analysed in biological triplicate, using individual plants and treatments to test for reproducibility. Following RNA isolation each of the samples was quantitated using a Nanodrop spectrophotometer. This provided the following information for each sample: concentration (ng/μl), the absorbance (A) in nm at 230, 260 and 280, the A_230_/A_260 _and A_260_/A_280 _ratios. Using this information the RNA yield and purity was calculated to ensure that they all had no significant impurities between samples that may affect reverse transcription and/or amplification during QRT-PCR. 1 μg of total RNA was reverse transcribed using the Bio-Rad^® ^(Sydney) iScript reverse transcription kit, according manufacturer's instructions. In parallel for each sample, another 1 μg of RNA was used for the same reverse transcription reaction, with the exception of the addition of the reverse transcriptase enzyme (no RT samples). Following this, the Qiagen^® ^PCR purification kit was used according to manufacturer's instructions on all samples (RT and "no RT" samples). This purified cDNA was diluted 1 in 10 with nuclease-free water and 1 μl was used for QRT-PCR analysis. For the no RT samples, no dilution was carried out and 1 μl was used in the same manner as the diluted cDNA for QRT-PCR analysis, this enables the detection of any genomic DNA contamination.

## Abbreviations

QRT-PCR: quantitative RT-PCR; SD: standard deviation; CV: coefficient of variance; MPSS: rice massively parallel signature sequencing; EF1d: elongation factor 1 delta; GAPDH: glyceraldehyde-3-phosphate dehydrogenase; HSF: heat shock factor; AOX: alternative oxidase; AP2: Apetela 2.

## Authors' contributions

RN carried out all the data analysis. RN, AI and SN carried out the experimental procedures. JW gave advice on the analysis, experimental procedures design and implementation. RN and JW drafted the manuscript. All authors read and approved final manuscript.

## Supplementary Material

Additional file 1 **Table S1**. List of genes analysed by QRT-PCR, primer sequences (5' to 3') and amplicon lengths (bp) are shown for each gene. Click here for file

Additional file 2 **Figure S1**. geNORM output using QRT-PCR data showing optimal number of reference genes required for accurate normalisation. Click here for file
