# Supplementary material for: Defining reference genes in Oryza sativa using organ, development, biotic and abiotic transcriptome datasets
Source: BMC Plant Biol. 2010 Mar 31;10:56. doi: 10.1186/1471-2229-10-56 (PMC2923530; doi:10.1186/1471-2229-10-56)
Supplement: Additional file 1 — Table S1. List of genes analysed by QRT-PCR, primer sequences (5' to 3') and amplicon lengths (bp) are shown for each gene. [file 1471-2229-10-56-S1.DOC]

**Table S1.** List of genes analysed by QRT-PCR, primer sequences (5’ to 3’) and amplicon lengths (bp) are shown for each gene.

| **TIGR Identifier** | **Description** | **Primer sequence (5' - 3')** | | **Amplicon** |
| --- | --- | --- | --- | --- |
| LOC_Os06g11170.1 | Nucleic acid binding protein | F: | ggaatgtggacggtgacact | 100 |
|  |  | R: | tcaaaatagagtccagtagatttgtca |  |
| LOC_Os06g43650.1 | Expressed protein | F: | ggtagacatcagtgccaggaa | 93 |
|  |  | R: | ctgagaggttccaacacaagc |  |
| LOC_Os06g47230.1 | Expressed protein | F: | gccgagaagaaggagtacga | 90 |
|  |  | R: | cttggccttaagctccttca |  |
| LOC_Os12g32950.1 | Membrane protein | F: | gagcgcaaagttccagaagaa | 164 |
|  |  | R: | cgccactagttgccgtcctgat |  |
| LOC_Os07g02340.1 | Expressed protein | F: | aggaacatggagaagaacaagg | 112 |
|  |  | R: | cagaggtggtgcagatgaaa |  |
| LOC_Os11g26910.1 | SKP1-like protein 1A | F: | gacgccgacttcgtcaag | 81 |
|  |  | R: | caaccccttgatgttgaggt |  |
| LOC_Os03g46770.1 | RNA-binding protein | F: | atgtcgagtaccgctgcttc | 120 |
|  |  | R: | tctccctgtcgttgatgatct |  |
| LOC_Os11g43900.1 | Tumor protein homolog | F: | cattggtgccaacccatc | 113 |
|  |  | R: | aaggaggttgctcctgaaga |  |
| LOC_Os11g21990.1 | Eukaryotic initiation factor 5C | F: | cacgttacggtgacacctttt | 90 |
|  |  | R: | gacgctctccttcttcctcag |  |
| LOC_Os07g34589.1 | Translation factor SUI1 | F: | gctgcaatggtactgttgtcc | 100 |
|  |  | R: | ccggcctgaacaagaaaat |  |
| LOC_Os05g48960.1 | Splicing factor U2af | F: | aggagccatgggagaagc | 79 |
|  |  | R: | gttcacggcggaaatcat |  |
| LOC_Os06g48970.1 | Protein kinase | F: | ggcgcttaaagaacttaagagga | 111 |
|  |  | R: | tgcatcgtagcccctgtaat |  |
| LOC_Os03g50890.1 | Actin | F: | ctcccccatgctatccttcg | 91 |
|  |  | R: | tgaatgagtaaccacgctccg |  |
| LOC_Os03g55270.1 | TIP41-like | F: | gtcatccggtcccacaac | 102 |
|  |  | R: | ctccccaaaaaccatctcag |  |
| LOC_Os08g23180.1 | Arabinogalactan protein | F: | gccaagaagctccaccag | 109 |
|  |  | R: | ggtgctgcgtgatgttca |  |
| LOC_Os07g42300.1 | Elongation factor 1-delta | F: | tggtgaggagactgaagagga | 114 |
|  |  | R: | ggtttgacatcaagcaacactg |  |
| LOC_Os01g39260.1 | FtsH protease | F: | gctgaaaacattgcagagagg | 76 |
|  |  | R: | aagattttctgaaacactaccaagg |  |
| LOC_Os03g25980.1 | Nucleotide tract-binding protein | F: | aacaacaagagccctggaga | 122 |
|  |  | R: | tgaacaaatccaaaagctgaga |  |
| LOC_Os02g46510.1 | AP-2 complex subunit | F: | tgcttgtgctttcaagtttgtc | 71 |
|  |  | R: | catcaaaagcaccaccaaag |  |
| LOC_Os03g21210.1 | endo-1,4-beta-glucanase | F: | ggtggtccagactacggtgt | 73 |
|  |  | R: | ttaaccggctcaaaagaacc |  |
| LOC_Os02g38920.1 | GAPDH | F: | tcattcctagcagcactggag | 80 |
|  |  | R: | agccattccagtcagctttc |  |
| LOC_Os06g46770.1 | Polyubiquitin | F: | cagcagcgcctcatcttc | 67 |
|  |  | R: | ggatgttgtagtcagccaagg |  |
| LOC_Os07g43730.1 | Elongation factor 1 | F: | aagaggaagtcagcggctaag | 77 |
|  |  | R: | cagaatgggcaggaaaataca |  |
| LOC_Os02g16040.1 | Ubiquitin | F: | ctcaaggacctgcagaagga | 94 |
|  |  | R: | atggacccatcagtgttgc |  |
| LOC_Os07g38730.1 | Alpha-tubulin | F: | tgttgattatggaaagaagtccaa | 94 |
|  |  | R: | gaggacactgttgtatggttctaca |  |
| LOC_Os05g36290.1 | Actin1 | F: | gtgattgcaccaccagaaag | 96 |
|  |  | R: | gcccttggagatccacatc |  |
| LOC_Os09g28440.1 | AP-2 | F: | gccaaagaaacggcaatg | 78 |
|  |  | R: | gcgacagagtcggagtcatc |  |
| LOC_Os04g01740.1 | HSF-82 | F: | ccctcatcatcaacaccttct | 95 |
|  |  | R: | ctcgaagcggatcttgtca |  |
| LOC_Os04g51150.1 | AOX | F: | gttccttgcaggcggagaag | 184 |
|  |  | R: | ccagtagtcgatggcgatgg |  |
